# The association of healthy eating index with periodontitis in National Health and Nutrition Examination Study 2011–2012

**DOI:** 10.3389/fnut.2022.999620

**Published:** 2022-09-26

**Authors:** Xin-yu Li, Hui Liu, Lu-yu Zhang, Xi-tao Yang

**Affiliations:** ^1^Department of Interventional Therapy, Multidisciplinary Team of Vascular Anomalies, Shanghai Ninth People’s Hospital, Shanghai Jiao Tong University School of Medicine, Shanghai, China; ^2^Department of Neurosurgery, Shanghai Ninth People’s Hospital, Shanghai Jiao Tong University School of Medicine, Shanghai, China; ^3^Department of Nephrology, Shanghai Jiao Tong University Affiliated Sixth People’s Hospital, Shanghai, China; ^4^The Department of Kidney Transplantation, The First Affiliated Hospital of Zhengzhou University, Zhengzhou, China

**Keywords:** periodontal disease, healthy eating index, periodontitis, dietary structure, NHANES (National Health and Nutrition Examination Survey)

## Abstract

**Aim:**

Periodontitis is a chronic inflammatory disorder caused by periodontopathic bacteria that causes inflammation of the supporting tissues around teeth. Previous studies have found that daily dietary nutritional intake can influence the development of periodontal disease. However, research on the Healthy Eating Index’s involvement in periodontitis is limited. The purpose of this study was to look at the link between the Healthy Eating Index and periodontitis.

**Methods and design:**

We examined data from the National Health and Nutrition Examination Study (NHANES), a nationally representative survey that was performed in 2-year cycles from 2011 to 2012. As part of our investigation, we used multivariate logistic regression models to investigate the independent relationship between the Healthy Eating Index and periodontitis. We used odds ratios (OR) with 95% confidence intervals to assess the significance of the connection (95% CI).

**Results:**

Individuals with a lower total healthy eating index were more likely to have periodontitis. A higher healthy diet index was associated with a lower prevalence of periodontitis (OR = 0.69; 95%CI: 0.49–0.97), according to adjusted multivariate regression models. The restricted cubic spline (RCS) analysis revealed that the non-linear relationship between HEI-2015 and periodontitis was statistically significant and that high HEI-2015 reduced periodontitis prevalence.

**Conclusion:**

The study’s findings revealed that dietary structure was linked to the prevalence of periodontitis. Patients with a higher Healthy Eating Index were less likely to have periodontitis. There is a need for future prospective longitudinal studies to confirm causality.

## Introduction

Periodontitis is an infection of the periodontium, a chronic inflammatory condition caused by microbes in the mouth (1). Periodontitis often leads to the destruction of periodontal support tissue and tooth loss and is known as the number one killer of oral health (2). Studies have shown that the pathogenesis of periodontitis is associated with dysbiosis of the resident oral flora, as well as an imbalance in the body’s immune and inflammatory response (3). Also, periodontitis is strongly associated with cardiovascular disease, cancer, diabetes, premature low birth weight babies, and respiratory infections (4). Previous research has shown that diabetes and metabolic syndrome, as well as genetic factors, all increase the risk of periodontitis (5, 6).

The Healthy Eating Index (HEI) is a method for assessing diet quality in accordance with the Dietary Guidelines for Americans (DGA) recommendations with a higher score indicating better compliance (7). Diet quality was assessed using the HEI-2015 scores, after adjusting data for demographic and lifestyle characteristics, and average nutrient intakes were obtained using the National Cancer Institute approach (8). The HEI-2015 is made up of 13 dietary components (7, 8). Total fruit, all fruit, all vegetables, greens and legumes, whole grains, dairy products, all protein foods, seafood and vegetable proteins, and fatty acids are all adequate components. Four moderate components include refined grains, sodium, added sugars, and saturated fats.

The concept that healthy eating habits promote health and unhealthy eating habits are linked to a variety of chronic diseases has gained widespread acceptance (9, 10). In previous studies, thiamine, riboflavin, niacin, and dietary inflammatory index were found to be associated with periodontitis (11, 12). However, research on the role of HEI in periodontitis is limited. The goal of this study was to compare the prevalence of periodontitis among participants with lower and higher HEI.

## Materials and methods

### Study population

NHANES is a nationally representative survey of the U.S. non-institutionalized population. Data were drawn from the NHANES 2011–2012.^1^ Full-mouth periodontal examinations were available to all adults aged 30 and up who had a permanent tooth (13). NHANES participants completed a questionnaire at home before undergoing a physical examination and interviews at a mobile exam center (MEC). The clinical examination data were standardized, with little site-specific bias. Because MEC examinations were only performed on a subset of NHANES participants, we only included those who reported a complete dental examination. Dietary quality was determined using 24-h dietary recalls and assessed using HEI-2015 (9, 10). We also included other demographic variables (including age, gender, race, education, smoking status and alcohol use status, diabetes, pre-diabetes, and physical activity) and BMI (body mass index). Ultimately, we included 3,055 participants for the next step of the analysis.

### Study variables

#### Socio-demographic characteristics

Socio-demographic characteristics were set as age, gender (male/female), race (Mexican American; white; black and other), education level (below high school; high school and college or above), smoking status (former; never and current), diabetes mellitus (DM; none; preDM), drinking status (never; former; light; moderate and heavy), and poverty income ratio (PIR). The following criteria are used to classify smoking status (14). A never smoker is defined as an adult who has never smoked or smoked less than 100 cigarettes in their lifetime; former smokers are those who reported smoking 100 cigarettes in their lifetime but were currently non-smokers; and current smokers are those who smoked 100 cigarettes on some days or every day in their lifetime. Never drinkers reported drinking less than 12 drinks; ever drinkers reported drinking more than 12 drinks in their lifetime but not in the previous year; and current drinkers were further classified as light, moderate, and heavy current drinkers. Heavy current drinkers were defined as women drinking 3 drinks per day and men drinking 4 drinks per day, with 5 or more binge drinking days per month; moderate drinkers were defined as women drinking 2 drinks per day and men drinking 3 drinks per day, with 2 binge drinking days per month. Light drinkers: did not meet the aforementioned criteria (15, 16). The poverty income ratio (PIR) is a metric that takes into account both reported income and household size. PIR was used to determine household income level, which is the ratio of family income to the appropriate poverty threshold: low (PIR < 1.35), medium (1.35 ≤ PIR < 3.0), and high (PIR ≥ 3.0). Diabetes is defined as the requirement for oral anti-diabetic medication or the administration of insulin (17). Prediabetes was defined as having high fasting glucose levels (18). Self-reported questionnaires were used to collect data on physical activity. A metabolic equivalent, or MET, is a unit of measurement that describes the energy expenditure of a particular activity (19). A MET is defined as the resting metabolic rate obtained while sitting still, set at 3.5 ml of oxygen consumed per minute per kg of body weight (20). NMET refers to an exercise that consumes N times more oxygen than sitting. The higher the MET, the more intense the exercise (21). The exercise habit was defined using a typical recording of weekly exercise activity in daily life. Levels of physical activity were converted into metabolic equivalent hours per week (MET/h/week). Metabolic Equivalent Hours = MET score * exercise time is the formula. Based on the participants’ MET, we divided them into quintiles (METQ1-Q5) (22). Underweight (BMI = 18.5), normal weight (BMI = 18.5–24.9), overweight (BMI = 25–29.9), and obese (BMI > 30.0) were the four BMI categories (23).

#### Periodontitis

A calibrated dentist assessed the participants’ periodontal status during the full-mouth periodontal examination. At the MEC, periodontal examinations included probing depths (PD) and clinical attachment levels (AL). The criteria for classification based on periodontal status are shown in Table 1 (24).

**TABLE 1:** Periodontitis classification criteria.

Classification	Standard
Mild periodontitis	≥2 interproximal sites with AL ≥3 mm, and ≥2 interproximal sites with PD ≥4 mm (not on same tooth) or one site with PD ≥5 mm
Moderate periodontitis	≥2 interproximal sites with AL ≥4 mm (not on same tooth), or ≥2 interproximal sites with PD ≥5 mm (not on same tooth)
Severe periodontitis	≥2 interproximal sites with AL ≥6 mm (not on same tooth) and ≥1 interproximal site with PD ≥5 mm
Noperiodontitis	No evidence of mild, moderate, or severe periodontitis

PD, Probing depths; AL, Clinical attachment levels.

##### Collection of dietary intake

NHANES dietary data is collected as part of the What We Eat In America survey (25). During the MEC visit, a 24-h dietary recall was conducted in-person with each NHANES participant in a separate room designated specifically for the dietary interview. The room where the interview took place was furnished with a computer loaded with Automated Multiple Pass software from the United States Department of Agriculture (USDA), food models, and three-dimensional measuring guides. These guides included glasses, bowls, mugs, mounds, circles, thickness sticks, spoons, a ruler, cartons, and five different sizes of water bottles. The MEC interviewer received specialized training to ensure that they could describe what to anticipate during the diet interview, explain the purpose of the diet recall, and ensure that they asked each participant the exact same questions, in the exact same way, while maintaining a completely objective demeanor (26). Both English and Spanish were options for the language of the interview. Individuals who were unable to report for themselves owing to factors such as age or disability had proxy interviews conducted on their behalf.

##### Healthy eating index

Other than fatty acids, each food component was evaluated per 1,000 kilocalories (kcals), with empty calories evaluated as a percentage of total kcals (27). The ratio of unsaturated to saturated fatty acids is used to express fatty acid content. Supplementary Table 1 shows the dietary components and scoring standards. The simple scoring method was used to calculate HEI, as this is the only calculation method that returns HEI values for individual participants. Higher scores for the adequacy components reflect higher quality. A lower intake will result in a higher score for the moderation components.

#### Statistical analysis

All analyses were performed using SAS v9.4 (SAS Institute) with *P*-value < 0.05 as a significant level. Data were presented as mean ± standard deviation for continuous variables and number (percentage) was used for the description of categorical variables. Using one-way analysis of variance and Chi-square tests, differences in the quantitative and qualitative characteristics at baseline for each HEI 2015 quartile were evaluated. The associations of HEI-2015 with periodontitis were estimated using binary logistic regression models with adjusting for potential confounders, in which the lowest quartile was used as the reference category. Model 1 adjusted for gender, age, and race. Model 2 additionally adjusted for education, DM, and PIR. Model 3 additionally adjusted for smoke, alcohol, and MET. The dose–response relationship between HEI-2015 and the risk of periodontitis was evaluated by a restricted cubic spline (RCS) as previously reported (28, 29). In addition, subgroup analyses were performed to evaluate the relationship between the HEI-2015 and periodontitis in different subgroups.

## Results

### Population characteristics

**FIGURE 1:**
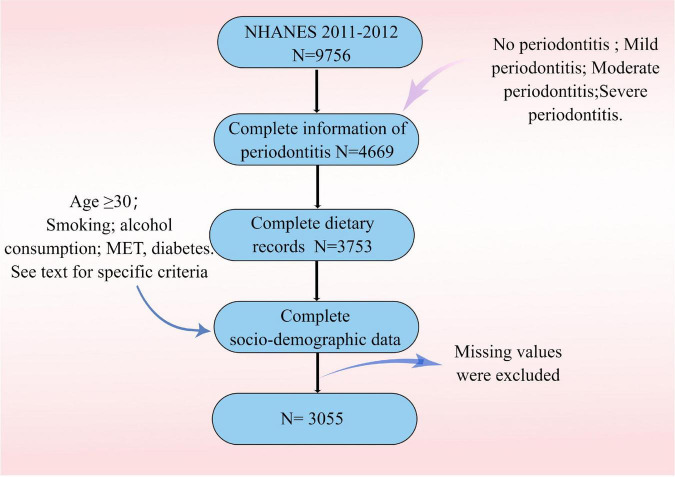
Flowchart of the study population.

Figure 1 depicts the recruitment and inclusion/exclusion criteria for the study. Periodontitis rates were statistically higher in several socio-demographic groups (Table 2). Participants with periodontitis had a higher mean age of 55.2 years than those without periodontitis (48.1 years). The prevalence of periodontitis is higher among whites and blacks, as well as among those with low economic incomes. The prevalence of periodontitis was also lower in participants with no history of smoking or alcohol consumption. The prevalence of periodontitis was also higher among diabetic and pre-diabetic participants than among non-diabetic participants. In the four HEI groups, the incidence of periodontitis showed a gradual decrease. Obese participants had the highest prevalence of periodontitis, but the difference was not statistically significant. Participants with the highest HEI scores had a lower prevalence of periodontitis than those with the lowest HEI scores (22.7 vs. 25.7%). In terms of periodontitis severity, participants in the highest quartile of HEI scores had a lower proportion of severe periodontitis than the other groups.

**TABLE 2:** Characteristics of the overall target population according to periodontitis.

Variables	Total (*n* = 3,055)	No periodontitis (*n* = 1,417)	Periodontitis (*n* = 1,638)	*p*
HEI2015Q4, *n* (%)				0.012
1	764 (25.0)	343 (24.2)	421 (25.7)	
2	763 (25.0)	331 (23.4)	432 (26.4)	
3	764 (25.0)	351 (24.8)	413 (25.2)	
4	764 (25.0)	392 (27.7)	372 (22.7)	
Age, Mean ± SD	51.9 ± 14.1	48.1 ± 13.6	55.2 ± 13.7	<0.001
Sex, *n* (%)				<0.001
Female	1,538 (50.3)	847 (59.8)	691 (42.2)	
Male	1,517 (49.7)	570 (40.2)	947 (57.8)	
Race, *n* (%)				<0.001
Black	780 (25.5)	282 (19.9)	498 (30.4)	
Mexican American	325 (10.6)	117 (8.3)	208 (12.7)	
Other Race	764 (25.0)	354 (25)	410 (25)	
White	1,186 (38.8)	664 (46.9)	522 (31.9)	
PIR_group, *n* (%)				<0.001
High	1,147 (40.5)	697 (51.9)	450 (30.2)	
Low	913 (32.2)	334 (24.9)	579 (38.8)	
Medium	774 (27.3)	311 (23.2)	463 (31)	
Education, *n* (%)				<0.001
Above high school	1,734 (56.8)	987 (69.7)	747 (45.6)	
High school graduate/GED or equivalent	644 (21.1)	229 (16.2)	415 (25.4)	
Less than high school	675 (22.1)	200 (14.1)	475 (29)	
Smoke, *n* (%)				<0.001
Former	760 (24.9)	326 (23)	434 (26.5)	
Never	1,720 (56.3)	928 (65.5)	792 (48.4)	
Now	573 (18.8)	163 (11.5)	410 (25.1)	
Alcohol.user, *n* (%)				<0.001
Former	506 (17.7)	181 (13.8)	325 (21.1)	
Heavy	516 (18.1)	192 (14.6)	324 (21.1)	
Mild	1,033 (36.2)	557 (42.3)	476 (30.9)	
Moderate	398 (13.9)	216 (16.4)	182 (11.8)	
Never	402 (14.1)	170 (12.9)	232 (15.1)	
preDM, *n* (%)				<0.001
DM	601 (19.7)	192 (13.6)	409 (25)	
No	1,508 (49.4)	814 (57.5)	694 (42.4)	
preDM	943 (30.9)	410 (29)	533 (32.6)	
BMI, *n* (%)				0.073
Normal weight	813 (26.8)	409 (29)	404 (24.9)	
Obese	1,130 (37.3)	502 (35.6)	628 (38.7)	
Overweight	1,056 (34.8)	483 (34.3)	573 (35.3)	
Underweight	33 (1.1)	15 (1.1)	18 (1.1)	
MET_group, *n* (%)				0.011
1	455 (19.7)	225 (20.1)	230 (19.4)	
2	462 (20.0)	227 (20.3)	235 (19.8)	

DM, diabetes; BMI, body mass index; MET, metabolic equivalent; preDM, prediabetes.

### Multivariate regression analysis

All four models revealed a negative relationship between the HEI-2015 and the prevalence of periodontitis based on the HEI quartiles (Table 3). According to the findings of the logistic regression analysis, higher quartiles of the HEI-2015 were associated with a lower prevalence of periodontitis. In model 1 (OR = 0.6; 95%CI: 0.48–0.75), model 2 (OR = 0.77; 95%CI: 0.6–0.98), and model 3 (OR = 0.69; 95%CI: 0.49–0.97), the prevalence of periodontitis was lower in the fourth quartile compared to the lowest quartile of the HEI-2015 population. *P* for trend was <0.05 in all models. A multivariate regression analysis of the HEI-2015 components revealed that the scores for whole fruits, whole grains, dairy, seafood, and plant proteins were all significantly related to periodontitis (Table 4). Supplementary Table 2 displays the results of subgroup analyses. Female (OR = 0.96; 95%CI: 0.94∼0.98); former smoker (OR = 0.97; 95%CI: 0.96∼0.98); moderate alcohol user (OR = 0.98; 95%CI: 0.96∼0.99); no diabetes (OR = 0.98; 95%CI: 0.97∼0.99); and METQ2 (OR = 0.97; 95%CI: 0.96∼0.98) were all associated with periodontitis.

**TABLE 3:** Association of HEI-2015 with periodontitis.

Exposure	Unadjusted Model	Model 1	Model 2	Model 3
HEI_Q1	1 (Ref)	1 (Ref)	1 (Ref)	1 (Ref)
HEI_Q2	1.06 (0.87∼1.3); 0.551	0.97 (0.78∼1.2); 0.757	1.07 (0.85∼1.35); 0.555	0.83 (0.58∼1.19); 0.312
HEI_Q3	0.96 (0.78∼1.17); 0.681	0.78 (0.63∼0.98); 0.029	0.88 (0.7∼1.12); 0.303	0.73 (0.51∼1.04); 0.08
HEI_Q4	0.77 (0.63∼0.95); 0.012	0.6 (0.48∼0.75); <0.001	0.77 (0.6∼0.98); 0.032	0.69 (0.49∼0.97); 0.033
*P*-value for trend	0.007	<0.001	0.035	0.039

Model 1: adjusted for gender, age, race.

Model 2: Model 1 + adjusted for education, DM, PIR.

Model 3: Model 2 + smoke, alcohol and MET.

DM, diabetes; BMI, body mass index; MET, metabolic equivalent; PIR, poverty income ratio.

**TABLE 4:** Association of HEI-2015 components with periodontitis.

HEI-2015 components	Unadjusted model	Model 1	Model 2	Model 3
Total vegetables	0.96 (0.92∼1.01); 0.086	0.94 (0.9∼0.98) 0.006	0.97 (0.92∼1.02) 0.21	0.97 (0.92∼1.02) 0.197
Greens and beans	0.98 (0.95∼1.02); 0.325	0.98 (0.95∼1.02) 0.278	1 (0.97∼1.04) 0.955	1 (0.97∼1.04) 0.847
Total fruit	0.97 (0.93∼1); 0.051	0.94 (0.9∼0.97) < 0.001	0.97 (0.93∼1.01) 0.11	0.97 (0.93∼1.01) 0.121
Whole fruits	0.96 (0.93∼0.99); 0.016	0.93 (0.9∼0.96) < 0.001	0.95 (0.92∼0.99) 0.012	0.95 (0.92∼0.99) 0.012
Whole grains	0.97 (0.95∼0.99); 0.002	0.95 (0.93∼0.97) < 0.001	0.96 (0.94∼0.99) 0.002	0.96 (0.94∼0.98) 0.001
Dairy	0.97 (0.95∼0.99); 0.001	0.97 (0.95∼0.99) 0.002	0.97 (0.95∼1) 0.022	0.97 (0.95∼1) 0.021
Total protein foods	1.02 (0.97∼1.09); 0.411	1.01 (0.95∼1.08) 0.674	1.04 (0.98∼1.11) 0.222	1.04 (0.97∼1.11) 0.285
Seafood and plant proteins	0.96 (0.93∼0.99); 0.008	0.95 (0.92∼0.98) 0.001	0.96 (0.93∼1) 0.04	0.97 (0.93∼1) 0.049
Fatty acids	1 (0.98∼1.02); 0.673	1 (0.98∼1.02) 0.791	1.01 (0.98∼1.03) 0.589	1.01 (0.98∼1.03) 0.59
Sodium	1 (0.98∼1.02); 0.944	1 (0.98∼1.03) 0.652	1 (0.98∼1.02) 0.934	1 (0.98∼1.03) 0.737
Refined grains	1 (0.98∼1.02); 0.9	0.99 (0.97∼1.01) 0.296	0.99 (0.97∼1.01) 0.242	0.99 (0.97∼1.01) 0.279
Saturated fats	1.02 (1∼1.04); 0.118	1.01 (0.99∼1.04) 0.254	1.02 (0.99∼1.04) 0.144	1.02 (1∼1.05) 0.09
Added sugars	1 (0.98∼1.03); 0.714	0.98 (0.96∼1) 0.113	1 (0.98∼1.02) 0.946	1 (0.97∼1.02) 0.718

Model 1: adjusted for gender, age, race.

Model 2: Model 1 + adjusted for education, DM, PIR.

Model 3: Model 2 + smoke, DM, alcohol and MET.

DM, diabetes; BMI, body mass index; MET, metabolic equivalent; preDM, prediabetes.

### Dose-response relationship between HEI-2015 and periodontitis

**FIGURE 2:**
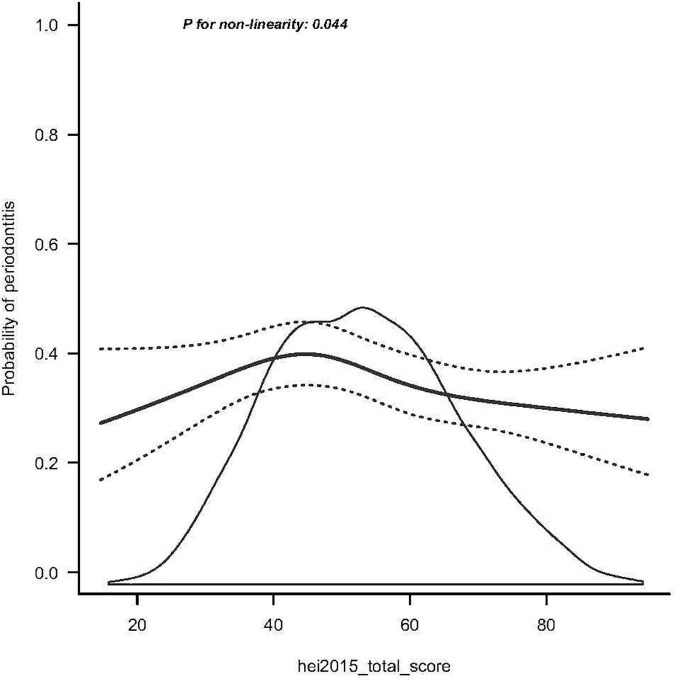
Smooth curve fitting of periodontitis and HEI-2015. The black solid line represents the average trend; black dotted lines correspond to the 95% credible interval.

The RCS analysis revealed a non-linear relationship between HEI-2015 and periodontitis after adjusting for potential confounders (Figure 2). As shown in Figure 2, the slope is steeper in the first third, and the subsequent lines continue to show a decreasing trend. This suggests that HEI-2015 is negatively related to periodontitis prevalence. Periodontitis can be reduced by increasing the HEI.

## Discussion

Our analysis revealed a significant correlation between a higher HEI score and a lower prevalence of periodontitis. Additionally, there is a connection between the severity of periodontitis and HEI. The prevalence of periodontitis was lower among participants with higher HEI scores than among those with lower HEI scores (22.7 vs. 25.7%). Periodontitis was less likely to affect those with higher dietary quality intake. Whole fruits, whole grains, dairy, seafood, and plant proteins were the ones most linked to periodontitis out of the 13 HEI components. These results suggest a link between periodontitis and diet (11).

The HEI has been evaluated and proven to be a reliable indicator of diet quality (11). The HEI consists of the following components: adequacy component (total fruit, whole fruit, total vegetables, greens and legumes, whole grains, dairy products, total protein foods, seafood and vegetable proteins, fatty acids) and moderation component (sodium, flavored grains, added sugars, saturated fats) (11, 12). Our data imply that the consumption of whole fruits, whole grains, dairy, seafood, and plant proteins is related with a decreased incidence of periodontitis. It has been shown that increasing one’s consumption of foods that are high in potassium, such as leafy green vegetables and fruits, may be helpful in lowering the risk of developing periodontitis (30) Omega-3 fatty acids seem to have a good impact on periodontal wound healing and may decrease periodontitis. Free fatty acids have a role in the development of periodontitis, and periodontitis may be reduced by omega-3 fatty acids (31). There have been fewer studies done on the relationship between periodontitis and plant proteins and seafood, but previous research has found that a daily intake of approximately one gram of calcium is beneficial for periodontal health and that there is a strong relationship between calcium intake and the development of periodontitis (32), with lower dietary calcium intake contributing to the incidence of periodontitis. Therefore, this further circumstantial evidence implies that a higher quality diet is related with a reduced prevalence of periodontitis, which is similar with the findings that we obtained.

People who smoke have a greater risk of developing chronic periodontitis, a more severe loss of alveolar bone, and larger periodontal pockets (33). Smoking is well acknowledged to be one of the most significant contributing factors in the development of chronic periodontitis (33). According to the findings of our study, the incidence of periodontitis was significantly greater in people who had a smoking history (current or former smokers). Quitting smoking was shown to reduce the risk of developing periodontitis. It is not known how smoking causes periodontitis, although it is thought to have a role in the disease’s progression. Smoking may prevent the antibacterial effect of the periodontal gingival sulcus fluid from working properly. Changes in the inflammatory components found in the gingival sulcus fluid are brought on by smoking, which ultimately results in an inflammatory response and structural damage to the periodontal tissue (33).

The present study is a large population-based cross-sectional investigation of the association between HEI and periodontitis. While previous research has investigated the link between individual nutrients and periodontitis (9, 10), the current investigation is of a more comprehensive nature. The HEI is a more thorough technique of nutritional evaluation and it is more indicative of an individual’s entire nutritional consumption. Therefore, conducting research into the connection between HEI and periodontitis may help paint a more complete picture of the dynamic between food intake and periodontitis. In addition, we come to the conclusion that the prevalence of periodontitis may be decreased by modifying the consumption of certain foods. We were able to further analyze the association between various kinds of food and periodontitis by doing research based on a multiple regression analysis of the 13 components that make up the HEI. However, some constraints remain. Due to the inability of cross-sectional observational studies to establish causality and directionality, our results should be regarded with caution. Despite thorough adjustments for confounding, residual confounding cannot be ruled out entirely. Due to the questionnaire’s retrospective character, there was a chance of memory bias among patients.

## Conclusion

Our findings suggest that periodontitis is more common among those with lower HEIs, indicating that dietary change is an important part of any health promotion program aimed at reducing the societal toll of periodontal disease.

## Data availability statement

The original contributions presented in this study are included in the article/Supplementary material, further inquiries can be directed to the corresponding author/s.

## Author contributions

All authors made substantial contributions to conception and design, acquisition of data, or analysis and interpretation of data; took part in drafting the article or revising it critically for important intellectual content; agreed to submit to the current journal; gave final approval of the version to be published; and agree to be accountable for all aspects of the work.

## Footnotes

1
http://www.cdc.gov/nchs/Nhanes

## Supplementary material

Supplementary Table 1 HEI–2015 components and scoring standards.

Supplementary Table 2 Subgroup analyses.
