# Supplementary material for: The association of healthy eating index with periodontitis in National Health and Nutrition Examination Study 2011–2012
Source: Front Nutr. 2022 Sep 26;9:999620. doi: 10.3389/fnut.2022.999620 (PMC9549051; doi:10.3389/fnut.2022.999620)
Supplement: Supplementary Table 2 — Subgroup analyses. [file Table_2.DOCX]

| **Subgroup** | **OR_95CI** | ***P*_value** | ***P*.for.interaction** |
| --- | --- | --- | --- |
| age≤30 | 1 (0.99~1.01) | 0.416 | 0.589 |
| age 30-60 | 0.99 (0.98~1) | 0.178 |  |
| age ＞60 | 0.99 (0.98~1) | 0.076 |  |
| sex=Female | 0.96 (0.94~0.98) | 0.047 | 0.041 |
| sex=Male | 0.99 (0.99~1) | 0.246 |  |
| PIR_group=high | 0.99 (0.98~1) | 0.087 | 0.468 |
| PIR_group=low | 1 (0.99~1.01) | 0.837 |  |
| PIR_group=medium | 1 (0.99~1.01) | 0.708 |  |
| smoke=former | 0.97(0.96~0.98) | 0.018 | 0.03 |
| smoke=never | 1 (0.99~1) | 0.441 |  |
| smoke=now | 0.99 (0.98~1.01) | 0.428 |  |
| alcohol.user=former | 0.99 (0.98~1.01) | 0.241 | 0.01 |
| alcohol.user=heavy | 1 (0.98~1.01) | 0.612 |  |
| alcohol.user=mild | 1 (0.99~1.01) | 0.501 |  |
| alcohol.user=moderate | 0.98 (0.96~0.99) | 0.004 |  |
| alcohol.user=never | 1 (0.98~1.01) | 0.899 |  |
| preDM=DM | 0.99 (0.98~1.01) | 0.338 | 0.02 |
| preDM=no | 0.98 (0.97~0.99) | 0.016 |  |
| preDM=preDM | 1 (0.99~1.01) | 0.929 |  |
| METQ1 | 1 (0.98~1.02) | 0.98 | 0.02 |
| METQ2 | 0.97 (0.96~0.98) | 0.021 |  |
| METQ3 | 0.99 (0.98~1.01) | 0.336 |  |
| METQ4 | 1 (0.99~1.01) | 0.97 |  |
| METQ5 | 0.99 (0.97~1.01) | 0.221 |  |

DM: diabetes; BMI: body mass index; MET: metabolic equivalent; PIR: poverty income ratio; preDM: prediabetes
